# A Multilevel Study of the Relationship between CSR Promotion Climate and Happiness at Work via Organizational Identification: Moderation Effect of Leader–Followers Value Congruence

**DOI:** 10.3390/ijerph19116637

**Published:** 2022-05-29

**Authors:** Jae-Geum Jeong, Seung-Wan Kang, Suk Bong Choi

**Affiliations:** 1 College of Global Business, Korea University, 2511 Sejong-ro, Sejong City 30019, Korea; j2gok@korea.ac.kr; 2 College of Business, Gachon University, Seongnam 13120, Korea

**Keywords:** team’s CSR promotion climate, leader–followers value congruence, organization identification, happiness at work

## Abstract

The purpose of the present study is to examine the effects of team level Corporate Social Responsibility (CSR) promotion climate on work happiness of team members. Furthermore, we investigate the mediating role of organizational identification at individual level and the moderating role of leader–follower value congruence at the team level in the relationship between CSR promotion climate and work happiness, thus overcoming the limitations of previous studies which mainly focused on a unitary level of analysis. To this end, a multilevel analysis was used, dealing with team- and individual-level relationships; the sample comprises 70 teams and 336 employees from 23 Korean firms. Our empirical analysis revealed that a team CSR promotion climate positively influenced team members’ happiness at work and organization identification. Furthermore, organization identification partially mediated the relationship between team CSR promotion climate and happiness at the workplace. By interacting with team CSR promotion climate, leader–followers value congruence positively regulated the influence of team CSR promotion climate on happiness at work. In this process, for a group with high leader–follower value congruence, the team CSR promotion climate strengthens team members’ happiness at the workplace. The study utilizes a multilevel analysis method to simultaneously verify team- and individual-level elements positively affecting team members’ happiness at work. Through this method, it confirmed that CSR promotion climate and team organization identification positively influence happiness at work. The theoretical and practical implications are presented, and directions for future research with limitations of the study are discussed.

## 1. Introduction

Corporate social responsibility (CSR) has been considered a strategic initiative as many companies are now adopting various co-prosperity strategiesto improve their sustainable competitiveness with internal and external stakeholders. They have also recognized the importance of internal human resources [1,2] as sources of competitiveness and sustainable growth of organization. In this vein, CSR activities have emerged as important approaches to enhance their global competitiveness and long survival [3,4]. In other words, CSR activities are perceived as essential means of generating profits—a company’s ultimate goal—and facilitating social consensus [5,6,7]. Moreover, companies need mutually reciprocal relationships with stakeholders for their sustainability and mutual growth which can enhance the company’s positive reputation, thereby serving as a key player of society [8]. Additionally, CSR activities are useful in improving the organization’s solidarity by motivating employees and broadening their understanding of the company’s goal and vision [9,10,11,12,13,14]. However, most studies on the effects of CSR have focused on issues relating to external stakeholders, such as consumers and local community perspectives, environmental protection, or philanthropic initiatives [12,15,16,17]. Thus, research on the role of internal stakeholders remains insufficient. Internal stakeholders are affecting and affected in various ways by the company’s CSR activities. Kim et al. [18] stated that CSR activities expand employees’ positive perceptions of their company’s vision and policies and improve their sense of pride and unity with their organization.

This sense of pride and unity can increase employees’ satisfaction with the organization, likely improving their happiness at work [19]. The happiness in the workplace can be a significant value and life goal for employees [20]. Previous literature reveals that when employees are happy, they are more creative in their work activities as well as have a higher level of identification with the organization and reduced turnover rate [21,22,23,24,25,26,27]. Therefore, employees’ happiness at the workplace can be seen as an important factor in increasing organizational effectiveness [26,27,28]. Therefore, employees who are happy at work can align their life goals to achieve organizational development. On the other hand, value congruence between leaders and followers, which make them share values, a moral foundation, and norm, affects employees’ level of leader–member exchange, job satisfaction, and organizational identification, eventually contributing to their organization and job performance [29]. The more congruent individual and leader values are, the more positively they affect organizational identification and job performance [30]. Organizational identification refers to the employee’s perception identifying with the organization’s vision, goals, and values that they are working for. Previous empirical studies have also revealed that organization identification is positively related to employees’ job performance and involvement in their work [31,32].

The CSR activities provide employees with a positive perception of the organization, enhancing trust and integrity with the organization [33]. Our study assumes that employees are the key internal stakeholders, playing an integral role in the company’s sustainable management. In addition, leader–followers value congruence can strengthen employees’ happiness, in conjunction with employees’ perception of CSR activities. Most behavioral scientists believe that social and environmental characteristics influence the members’ behavior [34,35,36,37,38,39]. Monson, Hesley, and Chernick [37] said that the effects of individual and situational characteristics on behavior should be considered simultaneously. Therefore, we investigate the role of the team CSR promotion climate and leader–followers value congruence as situational characteristics affecting employees’ work happiness. In sum, this study aims to conduct a multilevel analysis to understand the positive effects of CSR promotion climate at a team level on employee work happiness. We also explore the mediating role of organization identification to identify a mechanism connecting between them. Finally, the leader–followers value congruence’s moderating role on the relationship between team was also investigated.

## 2. Theoretical Background and Hypotheses

### 2.1. Team CSR Promotion Climate and Happiness at Work

The concept of CSR began to be discussed when it was first mentioned by Bowen [40]. Bowen set forth an initial definition of the social responsibilities of businessmen: “It refers to the obligations of businessmen to pursue those policies, to make those decisions, or to follow those lines of action which are desirable in terms of the objectives and values of our society” [41], p. 270. In addition, CSR was said to be an economic, legal, ethical, and philanthropic responsibility activity to pursue profits through honest and correct corporate activities and contribute to the community [42]. Additionally, the messages that organizational members receive from the organization concerning the type of behaviors that are important and that are expected, supported, and rewarded, are captured in the concept of organizational climate [43,44]. Therefore, this study defined Team CSR promotion climate as one in which CSR activities are positively recognized and encouraged by team members based on the concept of CSR in previous studies [41,42].

Specifically, team CSR promotion climate refers to the following aspects: (i) whether team leaders and members recognize and agree on the company’s pursuit of profits through honest and righteous activities; (ii) whether the economic, legal, ethical, and philanthropic responsibility activities contribute to the community [42]; and (iii) whether these activities are being encouraged within the team. CSR is obligated to follow plans that pursue ideal policies regarding society’s goals and values [45]. CSR activities are undertaken at the company level by strategic choice; they can also act as important guides in the work activities of individual members and working teams of an organization. For example, team members encouraging CSR can be willing to fulfill the economic aspects of product development and production and service activities provided to external stakeholders (consumers, suppliers, local communities and the media, countries, and governments) as well as their legal and ethical responsibilities. Such job performance depicts employees’ clear goals in job objectives, and their pride in the organization can increase [46].

On the other hand, happiness at work is positive feelings or emotions formed by satisfaction with jobs or organizations [19,47,48,49]. Diener et al. [48,50] said that it is a positive emotion formed by the perception that satisfaction with the organization and job helps realize one’s goals or ideals. Walter [49] said that he feels happy when he has the expectation that he can seek opportunities for learning and growth in his work. In addition, it was said that they find the meaning of life at work and have a sense of happiness through optimistic prospects for the future. Fisher [19] said that he has a sense of happiness through satisfaction with his job and positive emotional experience. Therefore, happiness at work can be said to be a positive feeling or emotion that satisfies one’s job and organization through positive experiences at work and predicts optimistically about the future. Diener [47] described happiness at the workplace as a multifaceted concept comprising life satisfaction as well as positive and negative emotions. Diener et al. [48] explained the concept of happiness through positive and negative emotions. In more specific terms, happiness at work entails a high level of employee satisfaction with the organization. Such satisfaction helps employees realize ideals and causes the employee to experience pleasant, positive emotions, enhancing their happiness [27,28,47,48]. Happiness intensity can be increased when the individual and the environment are integrated appropriately [19]. Therefore, happiness at the workplace is likely to be affected by specific environments, such as the goals and environment of the organization in which the employee works [51,52]. CSR activities comprise desirable values and goals, acting as social-responsibility initiatives for internal and external stakeholders. Therefore, employees who are a part of CSR activities gain social support, and their job is justified, thereby improving their satisfaction with the organization and their work [3,4]. As a result, they may experience positive emotions, improving their happiness at work. The team’s climate in which CSR is encouraged can increase members’ awareness, providing meaning for their job activities. A sense of job meaning is felt when the employees believe that the job entrusted to them fits their ideals and values [53]. Such a sense of meaning increases intrinsic motivation and job engagement as the employees commit themselves more passionately to the job [54]. Therefore, the employee’s sense of value during their job activities can help them realize standards that positively impact their happiness, increasing their sense of happiness at the workplace. CSR is also said to positively influence employees’ emotional engagement with the organization and civic-organizational behavior [55,56,57]. Fisher [19] claimed that employees who exhibit emotional engagement with the organization show attachment to the workplace, and such attachment triggers the positive emotions essential to happiness at work. Based on the above discussion, the following hypothesis was established.

**Hypothesis** **1.** *The team CSR promotion climate positively influences the team members’ happiness at work*.

### 2.2. Mediating Effect of Organizational Identification

Organization identification appears when members of the organization regard themselves as one with their organization and believe they are bound together by a shared destiny [58]. Organization identification is when an individual identifies with an organization and feels a sense of unity and belonging with the organization, becomes one with the organization by identifying themselves from the organization’s perspective, and regards the success and failure of the organization as their own [59,60].

Organization identification refers to an extension of an individual’s self to include their organization and relies on the organization for their sense of existence [46]. Ashforth and Mael [61] stated that organizational characteristics affect the concept of self of the organization’s members. Positive CSR perception and the team climate in which CSR is encouraged are also organizational characteristics. Team members believe they are a part of a good organization, inducing positive ego formation [61,62]. Consequently, they feel strong organization identification [63], and their happiness at work can increase through the development of positive perceptions and sentiments toward the team organization. Additionally, team characteristics that encourage CSR are stimulated and enhanced by the members’ organization identification. Moreover, a team climate that encourages CSR allows smoother communication than a team that does not. Smooth communication makes the members more passionate about their organization and increases organization identification through compensatory effects, such as self-respect [64,65]. Team members with a climate created and encouraged by desirable activities and values, such as CSR, have higher organization identification than members of teams that do not have such a climate [61]. Furthermore, Smidts et al. [65] stated that the more frequently an organization’s members are exposed to external sources of information about the organization, the more they feel the importance of their organization and themselves, attaching greater value to the organization and contributing to an increase in organization identification. Therefore, the climate of teams that encourage CSR can further increase organization identification through the perception of an exposure to positive aspects pertaining to the organization from external sources. Teams with such climates are likely to provide more desirable, higher-quality products and services to their work activities and stakeholders. Through such a climate and job activities, team members derive their reputation from external sources. A positive reputation can positively impact organization identification [66,67]. Furthermore, team members can derive a heightened sense of satisfaction from knowing that they, along with the organization, are being positively evaluated by external sources, facilitating an increase in happiness at work [47]. Members with organization identification are positive about their job satisfaction and professional involvement in their organizations [68]. Members’ job satisfaction and organizational involvement are achieved when their job aligns with organizational goals and values. This psychological state is where individuals reflect and identify their individual life goals or meaning that they add to the organization. Therefore, because team members develop their life meaning and goals through organization identification, their happiness at work can increase; organization identification enhances their task performance [69,70]. An enhancement in task performance can make the members feel satisfaction, reward, and a sense of accomplishment, increasing their level of happiness at work [26,28,47].

A team CSR promotion climate could be regarded as a dynamic activity that motivates team members and familiarizes them with the organization’s values and direction of objectives. Such a climate can be created based on clear communication and mutual trust between team members. Therefore, such a team climate can increase organization identification and positively increase happiness at the workplace. Organization identification felt by team members in such a relationship may be an important route between the team CSR promotion climate and happiness at work.

Therefore, the following hypothesis has been established.

**Hypothesis** **2.** *Team members’ organization identification positively mediates the relationship between team CSR promotion climate and happiness at work*.

### 2.3. Moderating Effect of Leader–Followers Value Congruence

Leader–followers value congruence refers to aligning the leader’s value with the followers [71,72]. This study proposes that this value congruence can be a boundary condition in the relationship between team CSR promotion climate and employees’ happiness at work. Individual values facilitate a broader understanding of the relationship between a leader and the followers [73,74]. Aligning individual values and the organization’s values positively affects the individual’s job satisfaction, loyalty, motivation, and task performance [75]. Additionally, members who share the organization’s values are highly likely to contribute to the organization constructively [29]. Value congruence is a belief that determines an individual’s attitude and behavior [76,77]. Such value congruence changes in the process of the member’s socialization, and value congruence is higher when an organization’s norms and values have strong characteristics [29,72]. Therefore, a team’s climate where CSR is encouraged can increase the leader–followers value congruence level.

Erdogan, Kraimer, and Liden [78] stated that value congruence quickly appears in the relationship between leaders and followers. Such a leader–follower relationship can enable smooth communication, create an amicable relationship [79], and positively influence the team climate that encourages CSR by heightening the level of positive perception and agreement of CSR. As value congruence is an essential factor influencing cohesiveness, which, in turn, affects individual happiness and the organization’s performance [80], value congruence is most likely to occur actively in the team CSR promotion climate. Chatman [29] stated that the higher the value congruence between individuals and an organization in an individual–organization fitting model, the higher the suitability between the individual and the organization. The leader–followers value congruence within a team can have high organization suitability between the two members. To say that members are highly suitable for the organization means the level of agreement and value congruence to the team climate that encourages CSR and the level of satisfaction with the job and the organization is high. Humans typically feel happier when they are in an environment that meets their particular needs or is consistent with their inclinations [81]. Members are happy at work through positive organizational experiences, and adverse experiences negatively affect their happiness at work [48]. In this context, the leader–followers value congruence as a mediator can positively influence the team climate that encourages CSR or happiness at work.

Through the above discussion, the following hypothesis was established.

**Hypothesis** **3.** *The leader–followers value congruence moderates the relationship between team CSR promotion climate and happiness at work. In other words, the influence of team CSR promotion climate on happiness at workis strengthened when the leader–followers value congruence is higher than when it is lower*.

**Figure 1:**
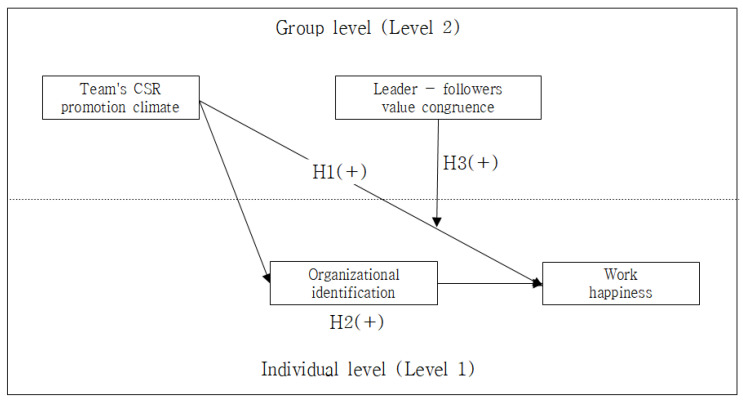
Study Model.

Figure 1 is the hypothesis model proposed in this study.

## 3. Methodology

### 3.1. Sample

As this study adopts a multilevel analysis method, individual and team levels should be considered simultaneously. Therefore, we selected a team organization with evident team characteristics and high interaction among team members. We surveyed the team members of 19 companies from January to June 2021. After obtaining permission from the managers, we distributed survey questionnaires and collected them individually through on-site visits. Further, a team-unit survey was conducted to increase the accuracy of responses. A total of 420 questionnaires were distributed. The survey respondents were 75 teams, and 374 surveys were collected (89.04%). In Korean companies, six months is generally considered the adaptation period necessary for job activities. This period is dedicated to learning about a job and adapting to the work environment. When job competency is established and adaptation is complete, employees are considered complete members of the team organization. Thus, those with less than six months of service in the team can be regarded as unstable members of the team organization and eliminated from the survey results. After eliminating incomplete responses or responses from employees with less than six months of work experience in the company, we collated 336 responses as the final data for analysis. Each of the final teams comprised a minimum of three to a maximum of eleven members; the average number of members per team was 4.8 (SD = 1.64).

The demographic characteristics of the respondents were as follows. There were more men (64.3%) than women (35.7%) among the respondents. The age distribution included respondents in their 30s (36%), who accounted for the largest portion, followed by those in their 40s (27.1%), 20s (19%), 50s (15.5%), and 60s (2.4%). Most of the respondents were college graduates (55.4%), followed by high school graduates (17%), junior college graduates (18.5%), master’s degree holders (8%), and Ph.D. degree holders (1.2%). The length of employment was evenly distributed from one to 30 years, with an average of 5.37 years (SD = 6.29). In terms of employment positions, general employees (38.4%) accounted for the largest portion of the sample, followed by assistant managers (21.4%) and managers (19.3%). The majority of the respondents were engaged in HR and general management (66.1%), followed by sales/marketing (15.2%), production (9.8%), and research and development (R&D) (8.9%).

### 3.2. Measures

This study’s variables were used by means of translating the scale from previous studies into Korean. After sufficient discussion with two professors in related fields, the scale was modified to be suitable for analyzing Korea’s situation and team level [82]. A 5-point Likert scale was used for all items; 1 point represented “strongly disagree,” while 5 represented “strongly agree”. The definition and measurement items of key variables are provided in Appendix A.

#### 3.2.1. Team CSR Promotion Climate

Team CSR promotion climate measures the degree to which the team leaders and members perceive and agree with their company’s economic, legal, ethical, and philanthropic responsibility activities to pursue profits through honest and proper business activities and contribute to the local community [42], and the degree to which these activities are promoted within the team. Of the 29 items used in Maignan et al. [42], this study modified and employed five items each, from economic, legal, ethical, and philanthropic activities. The CSR team members’ promotion climate scores, including team leaders, were aggregated and averaged for use as a group variable. The reliability coefficient (Cronbach’s alpha), representing internal consistency among items, was 0.95. Representative items included “My team endeavors to establish a procedure to respond to all customer complaints” and “My team strives to comply with legal standards for products and services”.

#### 3.2.2. Organizational Identification

Organizational identification refers to the individual’s cognitive perception of oneness and shared destiny with an organization to which they belong [58]. This study employed six items used in Mael and Ashforth [58,83]. The reliability coefficient (Cronbach’s alpha), representing internal consistency among items, was 0.86.

Representative items included “I feel as if I am insulted when someone criticizes my company” and “I use the expression ‘we’ when describing my colleagues”.

#### 3.2.3. Leader–Followers Value Congruence

This study used Cable and DeRue’s [71] measure—which comprises three items and is a modification of Cable and Judge’s measure developed in 1996 [84]—to measure leader–followers value congruence. Previous studies had measured organizations and individuals as the object of value matching; however, this study modified the organization into a team leader because the organization represents a team leader and an individual. The leader–followers value congruence scores of team members, including those of team leaders, were aggregated and averaged for use as a group variable. The reliability coefficient (Cronbach’s alpha), representing the internal consistency among items, was 0.91. The sample items used in this study included “My personal values are consistent with those of my leader” and “Values pursued by my leader are highly similar to my values”.

#### 3.2.4. Happiness at Work

Happiness at work is a multifaceted concept that can be subdivided into life satisfaction, positive emotion, and negative emotion [47]. We employed the Satisfaction with Life Scale (SWLS) developed by Diener et al. [60] to measure life satisfaction, whereas Diener et al.’s [48] Scale of Positive and Negative Experience (SPANE) measured positive and negative emotions, which represent emotional happiness. This measure was originally developed to determine general happiness in life; this study modified the measure to determine happiness at work. The measure, comprising nine items, was subdivided into three groups consisting of three items to measure life satisfaction, positive emotion, and negative emotion. A 5-point Likert scale, ranging from 1 point (strongly disagree) to 5 points (strongly agree), was used to measure happiness at work, and the scores of the third item were inversed. The reliability coefficient (Cronbach’s alpha), representing the internal consistency among items, was 0.94.

Representative items included “I am satisfied with my company” and “My work life has mostly been pleasant”.

### 3.3. Data Analysis

A multilevel analysis was used to examine group- and individual-level antecedent factors affecting employees’ happiness at work and the mediating effect of situational variables. Hierarchical Linear and Nonlinear Modeling (HLM) 7.01 was used as previous studies in organizational behavior and leadership commonly have applied [85,86,87,88,89,90]. As this study obtained both independent (team’s CSR promotion climate) and dependent (happiness at work) variables from the same source during the same time frame, there is a possibility that common method bias may occur [91]. Therefore, whether a particular factor accounts for most of the overall variance was examined as a follow-up measure. It was found that three factors had an eigen value of 1.0 or higher. The variance ratio of the factor with the largest eigen value was 28.2%, indicating a low possibility of common method bias.

As this study’s measurement tool was developed based on the subjective responses of team members, confirmatory factor analysis (CFA) was used to examine construct and discriminant validity. Specifically, four research models, comprising a team’s CSR promotion climate, organizational identification, leader–followers value congruence, and happiness-at-work factors, were analyzed and compared (Table 1). The CFA results indicate that the values of χ^2^(df), CFI, TLI, IFI, RMR, and RMSEA of the four-factor (proposed) model were 1220.86 (647), 0.94, 0.93, 0.94, 0.03, and 0.05, respectively. In addition, NFI, AGFI, and SRMR values were 0.88, 0.89, and 0.05, respectively. The results of the χ^2^ test also showed that the four-factor model had better suitability of fit than alternative models. These results support the construct and discriminant validity of the measurement.

Additionally, the discriminant validity of group variables, a team’s CSR promotion climate and leader–followers value congruence were examined based on the r_wg_ index of agreement and the ICCs index of reliability. If the r_wg(j)_ value is 0.70 or higher, the aggregated data can be used as group-level data. The ICC (1) typically ranges from 0.05 to 0.20, and an ICC (1) greater than 0.30 can be seen as highly desirable [92]; an ICC (2) between 0.50 and 0.70 can be partially accepted, and an ICC (2) over 0.70 can be seen as desirable [93].

Table 2 presents the analysis results. All values exceeded the appropriate standard; however, the ICC (2) for leader–followers value congruence was slightly lower than 0.70, indicating partial acceptance [92,94,95]. The results of the *F*-test were also significant, justifying the analysis of a team’s CSR promotion climate and leader–followers value congruence as group variables. These results support the discriminant validity of the group variables in the multilevel analysis.

## 4. Result

### 4.1. Descriptive Statistics and Correlations

This study conducted a correlation analysis to investigate the relationship between measurement variables. As depicted in Table 3, four variables were correlated in the proposed research model. Happiness at work, the individual-level dependent variable, was positively correlated with organizational identification (individual-level variable; *b* = 0.61, *p* < 0.01), a team’s CSR promotion climate (group-level variable; *b* = 0.58, *p* < 0.01), and leader–followers value congruence (group-level variable; *b* = 0.61, *p* < 0.01). A team’s CSR promotion climate, the group-level independent variable, was positively correlated with leader–followers value congruence (group-level variable; *b* = 0.57, *p* < 0.01) and organizational identification (individual-level mediating variable; *b* = 0.52, *p* < 0.01).

### 4.2. Hypotheses Test

If a dependent variable is completely explained at the individual level, additional group-level variables are unnecessary for the research model. Based on this, we examined the variance of the dependent variable, employees’ happiness at work, which cannot be explained at the individual level. As shown in Table 4, significant group-level variance (intercept) was found in the random effect of the null model, confirming the necessity of the multilevel analysis (group-level variance *τ* = 0.14, *p* < 0.001). The calculated ICC of the null model was 33.2% [0.14/(0.29 + 0.14)], indicating that, although 33.2% of the dependent variable’s total variance cannot be explained by individual-level variables, it can be elucidated through group-level variables. This observation clarifies that individual-level factors explain 66.8% of employees’ happiness at work; however, the remaining 33.2% can be explained by group-level factors. Therefore, a multilevel analysis was employed to test this study’s research hypotheses, including individual- and group-level factors.

A hierarchical regression analysis was conducted to test Hypotheses 1 through 3 (Table 4). Hypothesis 1, “a team’s CSR promotion climate can positively affect employees’ happiness at work”, was tested, holding gender, age, and education level constant (Model 2). A statistically significant effect was found (*b* = 0.48, *p* < 0.001), therefore, Hypothesis 1 was supported.

Hypothesis 2, “Organizational identification can positively mediate the relationship between a team’s CSR promotion climate and employees’ happiness at work”, was also tested. A team’s CSR promotion climate, a group-level variable, positively affected organizational identification, an individual-level variable (*b* = 0.39, *p <* 0.001). As depicted in Model 2, organizational identification and individual-level variable positively affected, employees’ happiness at work, which is the dependent variable (*b* = 0.50, *p <* 0.001). Overall, a team’s CSR promotion climate directly affected employees’ happiness at work, and the mediating effect of organizational identification was also significant. The organizational identification variable played a role as a partial mediator, and Hypothesis 2 was supported.

**Figure 2:**
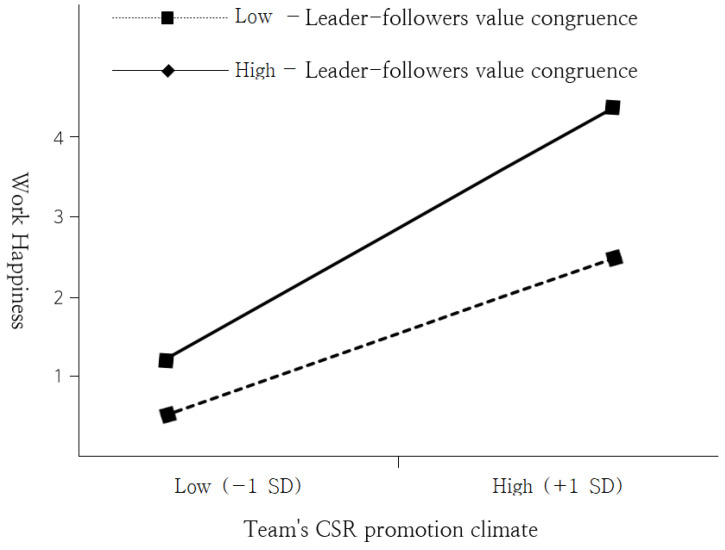
Moderating effect of leader–followers value congruence.

We also tested Hypothesis 3: “The leader–followers value congruence can moderate the relationship between team CSR promotion climate and happiness at work. In other words, the influence of team CSR promotion climate on happiness at work can be strengthened when the leader–followers value congruence is higher than when it is lower”. In other words, we tested whether group-level interaction mediates the effect on an individual-level dependent variable, employees’ happiness at the workplace. All of the research model’s variables, including both individual- and group-level, were included in Model 3. The interaction between a team’s CSR promotion climate and leader–followers value congruence was also included. The interaction between a team’s CSR promotion climate and leader–followers value congruence positively affected employees’ happiness at work (*b* = 0.25, *p <* 0.001). The mediating effects for both the high leader–followers value congruence level (M + 1SD) and low leader–followers value congruence level (M-SD) were indicated as slopes in Figure 2 [96] to facilitate the interpretation of these results.

The effects of high congruence level (*t* = 2.194, *p <* 0.05) and low congruence level (*t* = 2.093, *p <* 0.05) on the relationship between a team’s CSR promotion climate and employees’ happiness at work were significant; however, a high congruence level significantly strengthened the effect of a team’s CSR promotion climate on employees’ happiness at work. Therefore, Hypothesis 3 was also supported.

## 5. Discussion and Implications

As corporate are required to contribute to Society’s interest and improvement, CSR has received much attention from many academic researchers, managers, and policy makers in various field. CSR in economics is viewed primarily as a private provision of public goods [97] and argues that it imposes sacrifices for impossible gains in competitive markets [98]. Thus, many studies in economics are concerned with the economic aspect of whether CSR activities can maximize corporate profits [99]. It is interested in whether CSR activities in financial research have a positive effect on corporate financial performance. These studies have been focused on the overall relationship with corporate governance for successful CSR [100]. CSR activities in the field of organization studies have focused on the motivation or behavior of members of the organization to promote CSR [101,102]. Employees argued that they were very interested in the organization’s evolving social consciousness and that a clear judgment of the organization’s CSR activities efforts determines its subsequent attitudes and behaviors of employees [101]. However, as most previous studies in the area of CSR were focused on macro-level factors with CSR activities, studies on micro-level activity exploring individual factors affecting CSR activity and its outcome have been limited. Therefore, this study focused on the role of corporate CSR promotion climate for determining employees’ attitudes and behaviors toward their work at the micro-level.

This study deepened the understanding of a company’s corporate social responsibility (CSR) activities and confirmed that the organization’s climate significantly influences the individual. Team CSR promotion climate was an important antecedent influencing employees’ happiness at work, indicating that when the organization’s intended values or goals are encouraged in a unit organization, such as a team, a happy workplace is created for each member. The influence of organization identification on member and organizational well-being has been long recognized [103]; it has been established that team CSR recognition climate is an antecedent for such organization identification. In this relationship, the mediation model of organization identification acts as an essential factor influencing employees’ happiness at work in a climate where CSR is encouraged. Furthermore, this study confirms that the higher the leader–followers value congruence, the more the workplace happiness is positively affected by the team CSR promotion climate. In particular, this study confirmed that team CSR promotion climate further strengthens happiness at work when the leader–followers value congruence was higher as opposed to when it was lower. Thus, creating a climate in which CSR is emphasized and encouraged can increase employee happiness at work, which is essential to the company’s sustainable growth and development. Such findings have theoretical implications in that they expand the existing body of literature on CSR and employee happiness at work. They also have considerable practical implications in creating a climate within a company’s organization.

### 5.1. Theoretical Contributions

First, most studies on CSR activities were from the external stakeholder perspective [12,13,16,104], and studies on the internal stakeholder perspective were relatively sparse. This study hopes to overcome these limitations and expand the scope of research by addressing the positive impact of CSR on internal stakeholders, such as team leaders and team members, and on employees’ happiness at work.

Second, most CSR studies focused on a single level, such as company level or individual level; however, members of the organization can acknowledge CSR as values or climates toward which the organization can aim. While social and environmental characteristics influence the behavior of individual members [34,35,36], it is challenging to accurately analyze the influential relationship on individual members through research at a single level. Therefore, for a multilevel analysis, this study used team-level variables, such as team CSR promotion climate and leader–followers value congruence, and demonstrated the need for theoretical expansion and multilevel analysis by proving the influential relationship between the team and the individual.

Third, this study identified that a team climate encouraging CSR is an important variable that positively influences employees’ happiness at work. Organization identification serves as an important route in this relationship. A team climate for value congruence is a mechanism that moderates this relationship.

This study deepened and expanded the understanding of this relationship by simultaneously considering the relationship between CSR and employee happiness at work, which have been recent topics of interest.

### 5.2. Managerial Implications

First, considering that CSR is a significant source of competitiveness in a company’s sustainable growth and development [8], this study proved that the CSR promotion climate of unit team organizations could play a pivotal role in team members’ happiness at work. Internal human resources are key sources of competitiveness [1,2]; if team members are happy at work, their positive states of mind can positively influence their job performance [29]. Therefore, companies and managers should pay more attention to and be more inclined toward encouraging CSR to stimulate team members’ motivation, broaden the organization’s understanding, and spread positive perceptions. For example, organizations should actively encourage their employees to recognize that the company’s CSR activities are desirable in terms of social goals or values. The company could also establish policies to clarify the employees’ perception of CSR and apply CSR in their work activities.

Second, this study showed that organization identification is a vital recognition mechanism in employee happiness at work. This study verified that team CSR promotion climate is a significant antecedent in employee happiness at work and that organization identification is an essential factor in this process. Organization identification appears when the organization’s members positively perceive the organization and believe that they share a common destiny with the organization [61]. As a result, the members’ satisfaction with the organization increases, improving task performance and happiness at work [61,68,69]. Therefore, to create a team CSR promotion climate, companies can educate and train team leaders to more actively improve and enhance the climate in the team.

Third, this research suggested that high leader–followers value congruence is more effective in facilitating happiness at work for employees who are positive about their job performance and the organization’s development. This study also showed that team CSR promotion climate and leader–followers value congruence are important in increasing employees’ happiness at work. Erdogan, Kraimer, and Liden [78] stated that value congruence quickly appears in the relationship between leaders and followers; however, leaders must check their perception of CSR vis à vis that of their team members. Aligning CSR perceptions can be a meaningful effort and action for the leader to improve value congruence. Ultimately, increasing the leader–followers value congruence can result in the team CSR promotion climate heightening employees’ happiness at work.

In addition to the empirical analysis of our research, we examined two cases in the Korean manufacturing industry that led to a successful financial and social performance through CSR activities. The two cases include the Pulmuone and Yuhan manufacturing companies. Pulmuone and Yuhan have long used CSR as a corporate value and motto. Pulmuone is a food and beverage manufacturing firm. It is a medium enterprise that produces the right healthy food with the aim of improving the health of customers, the environment, and eventually the society it is operating within [105]. Pulmuone makes shared value creation, product safety, quality control, customer satisfaction, communication, and environmental protection their keyissues of CSR [106]. Pulmuone’s business philosophy is to implement a LOHAS (i.e., Lifestyle of Health and Sustainability) life based on the spirit of “neighbor-love” and “life-respect”. The founder’s business philosophy and leadership were created as a climate by spreading a positive perception of CSR around team leaders and led to active participation in CSR by members of the organization. As a result, external stakeholder believes in Pulmuone and Pulmuone’s product through positive perceptions of Pulmuone. Moreover, employees became proud of the organization and motivated to lead to high performance and satisfaction. Pulmuone is continuously growing and has been selected as the “The First Korea’s Most Admired Companies” for 15 consecutive years as of 2022 [107]. Pulmuone’s CSR promotion climate and shared values increased the sense of unity within the organization and contribute to feeling happy at work.

Secondly, Yuhan is a medium pharmaceutical manufacturing enterprise. Yuhan’s business philosophy is to gain profit through sincere business activities and return a profit to society. AsCSR was created in the environment surrounding the firms [108], Yuhan CSR activities are based on business principles that value compliance management, social contribution, and environment and safety. Yuhan has established an internal compliance system and principles of activity to comply with the fair trade act. These are means that firms must return profits earned to the society which raised the firms. Yuhan’s CSR goal contributed to national health and employee welfare so that all employees could build a firm culture of a group bound together by a common destiny [106]. As a result, the perception of CSR spread to organizations, and shared values were created. This climate led to employees’ perception that employees regarded their organizational identification with pride.

Happiness at work can be seen as starting from pride in the organization and the job. Pulmuone and Yuhan gained a high social reputation through CSR activities as the business philosophy and goal. Moreover, both firms have established themselves as happy workplaces by not only creating a healthy society where firms belong, but also by gaining high support, loyalty, and pride. The above two Korean firms have created workplace happiness where active CSR activities and the CSR promotion climate help employees match the values and increase organizational identification to make their lives rewarding and proud. Thus, Pulmuone and Yuhan CSR activities success cases supported this research results.

### 5.3. Limitations and Future Research Direction

Despite the above contributions, this study has several limitations, suggesting future research directions.First, this study has limitations in identifying the causal relationships presented in the research model; data collection comprises cross-sectional collection and may have a common method bias. Therefore, with data collection through longitudinal design and experiments being conducted in this area in the future, a more accurate research result may be derived. Furthermore, measuring recognition-based responses may reduce the potential perception bias. Second, although leader–followers value congruence and organization identification have been verified in the positive influential relationship between promoting team CSR climate and employees’ happiness at work, various factors influence employee perception and values of happiness. Therefore, future research should consider leadership style and the characteristics of leaders’ and employees’ personalities in the relationship for a more valuable result.

## 6. Conclusions

Using a multilevel analysis model tackling team- and individual-level factors simultaneously, we empirically investigated how and under what conditions a team’s CSR promotion climate can influence team members’ happiness at work. This study’s findings confirmed a positive effect of a team’s CSR promotion climate on team members’ happiness at work, demonstrated the significant mediating roles of organizational identification. They also indicate a positive moderating role of leader–follower value congruence in their relationship. Despite this study’s limitations, this finding has important implications for organizations aiming to improve team members’ happiness at work, a key source of sustainable development in today’s business environment.

## Figures and Tables

**Table 1:** Comparison of measurement models.

Model	*χ*^2^ (*df*)	CFI	TLI	IFI	RMR	RMSEA
Four-factor(proposed) model (CSR, OI, VC, WH)	1220.86 (647)	0.94	0.93	0.94	0.03	0.05
Three-factor model (WA & VC merged, OI, WH)	1658.95 (650)	0.89	0.88	0.89	0.04	0.06
Two-factor model (WA & VC merged, OI & WH)	3297.37 (663)	0.73	0.71	0.73	0.05	0.10
One-factor model	4583.97 (665)	0.60	0.57	0.60	0.06	0.13

Notes: CSR= Team’s CSR promotion climate; OI = Organizational identification; VC = Leader–followers value congruence; and WH = Work happiness.

**Table 2:** Aggregation Test Results for Group-Lever Variables.

Variables	r_wg(j)_	ICC (1)	ICC (2)	F (*p*-Value)
Team’s CSR promotion climate	0.98	0.44	0.79	4.82 (*p* < 0.001)
Leader–followers value congruence	0.88	0.25	0.62	2.63 (*p* < 0.001)

Notes: r_wg(j)_ = Average interrater reliability; ICC (1) = Interclass Correlation Coefficients assessing the interrespondent reliability; ICC (2) = Interclass Correlation Coefficients assessing the mean reliability of a group.

**Table 3:** Descriptive Statistics and Correlations.

(a) Individual(Level 1) Variables		Mean	SD	1	2	3	4	5	6	7	8
1.	Gender	1.35	0.47	-							
2.	Age	2.46	1.04	−2.23 **	-						
3.	Education	2.58	0.90	−0.18	−0.21 **	-					
4.	Organization identification	3.92	0.62	−0.02	0.29 **	0.06	(0.86)				
5.	Happiness at work	3.82	0.65	−0.05	0.11 *	0.12 *	0.58 **	(0.94)			
**(b) Team(Level 2) Variables**											
6.	Team size	5.36	1.99	0.30 **	−0.12 *	−0.02	−0.00	0.04			
7.	Team’s CSR promotion climate	3.88	0.60	−0.00	0.06	0.17 **	0.52 **	0.61 **	0.01	(0.95)	
8.	Leader–followers value congruence	3.53	0.74	−0.00	0.07	0.18 **	0.61 **	0.61 **	0.01	0.57 **	(0.91)

**Notes**: N = 336 for level-1 variables and 70 for level-2 variables. Values in parentheses are alpha coefficients. * *p* < 0.05; ** *p* < 0.01, Two-tailed tests; SD = standard division.

**Table 4:** Hierarchical linear model predicting: direct, mediating, and moderating effects.

Variables	Organization Identification	Happiness at Work			
Model		Null Model	Model 1	Model 2	Model 3
**Individual Level**					
Intercept	3.90 ***	3.811 ***	3.844 ***	3.84***	3.83 ***
Gender	–0.01		−0.098	−0.10	−0.08
Age	0.21 ***		−0.043	−0.05	−0.05
Education	0.03		−0.004	−0.00	−0.00
Organization identification			0.503 ***	0.50***	0.50 ***
**Group level**					
Team size	−0.00			0.02	0.03
Team’s CSR promotion climate	0.39 ***			0.48***	0.50 ***
Leader–followers value congruence				0.36***	0.34 ***
**Group level interaction**					
Team’s CSR promotion climate × Leader–followers value congruence					0.25 ***
**Random effect**	Variance component	Variance component	Variance component	Variance component	Variance component
Group-level variance(τ)	0.0511 **	0.1460 ***	0.1928 ***	0.0244 ***	0.0161 ***
Individual-level variance(σ2)	0.2449	0.2935	0.2073	0.2007	0.1995
Deviance	578.66	626.52	563.51	496.59	491.58
*χ* ^2^		224.28	23.61	22.18	22.20

**Note**: ** *p* < 0.01; *** *p* < 0.001.

## Data Availability

Not applicable.

## Appendix A. Definition and Questionnaire Items

**Table A1:** Team’s CSR promotion climate.

**Definition**	Team leaders and members recognize, agree and are encouraged within the team to pursue profits through honest and correct corporate activities and engage in economic, legal, ethical and philanthropic responsibility activities to contribute to the community.
**References**	Maignan, I., Ferrell, O. C., & Hult, G. T. M. (1999). Corporate citizenship: Cultural antecedents and business benefits. Journal of the Academy of Marketing Science, 27(4), 455–469. [42]
**Questionnaire Items**	***Economic Citizenship*** Our business has a procedure in place to respond to every customer complaint. We continually improve the quality of our products. We use customer satisfaction as an indicator of our business performance. We strive to lower our operating costs. Top management establishes long-term strategies for our business. ***Legal Citizenship*** Managers are informed about relevant environmental laws. All our products meet legal standards. The managers of this organization try comply with the law. Our company seeks to comply with all laws regulation hiring and employee benefits. Internal policies prevent discrimination in employees’ compensation and promotion. ***Ethical Citizenship*** Members of our organization follow professional standards. Top managers monitor the potential negative impacts of our activities on our community. We are recognized as a trustworthy company. Fairness toward coworkers and business partners in an integral part of our employee evaluation process. Our salespersons and employees are required to provide full and accurate information to all customers. ***Discretionary Citizenship*** Our business encourages employees to join civic organizations that support our community. Our business gives adequate contributions to charities. A program is in place to reduce the amount of energy and materials wasted in our business. We encourage partnerships with local businesses and schools. Our business supports local sports and cultural activities.

**Table A2:** Organizational Identification.

**Definition**	A member of an organization perceives himself and the organization as a concept and considers it a common destiny.
**References**	Mael, F. A., & Ashforth, B. E. 1992. Alumni and their alma mater: A partial test of the reformulated model of organizational identification. Journal of Organizational Behavior, 13(2), 103–123. [83] Mael, F. A., & Ashforth, B. E. 1995. Loyal from day one: Biodata, organizational identification, and turnover among newcomers. Personnel Psychology, 48(2), 309–333. [58]
**Questionnaire Items**	When someone criticizes (name of company), it feels like a personal insult. I am very interested in what others think about (name of company). When I talk about this company, I usually say “we rather than ‘they’ This company’s successes are my successes. When someone praises this company, it feels like a personal compliment. If a story in the media criticized the company, I would feel embarrassed.

**Table A3:** Leader–followers value congruence.

**Definition**	It means that the values of the leader and followers match.
**References**	Cable, D, M, & Judge, T, A, (1996), Person-organization fit, job choice decisions, and organizational entry. Organizational Behavior and Human Decision Processes, 67, 294–311. [84]
**Questionnaire Items**	The things that I value in life are very similar to the things that my team leader values. My personal values match my team leader’s values and culture. My team leader’s values and culture provide a good fit with the things that I value in life.

**Table A4:** Happiness at Work.

**Definition**	Happiness at work refers to a positive feeling or emotion that satisfies one’s job and organization through positive experiences at work and predicts optimistically about the future.
**References**	Diener, E., Larsen, R. J., & Emmons, R. A. (1984). Person × Situation interactions: Choice of situations and congruence response models. Journal of Personality and Social Psychology, 47(3), 580–592. [81] Diener, E., Emmons, R. A., Larsen, R. J., & Griffin, S. (1985). The satisfaction with life scale. Journal of Personality Assessment, 49, 71–75. [50]
**Questionnaire Items**	I’m satisfied with the job I belong to. I am satisfied with the conditions of my job. My job is very helpful in realizing the ideal of my life. My working life was generally happy. My work life was generally enjoyable. My work life was generally positive. My work life was generally unpleasant. My work life was generally sad. My work life was generally upsetting.
